# A lightweight soil moisture prediction model based on irrigation cycle segmentation and Kalman filtering

**DOI:** 10.3389/fpls.2026.1853119

**Published:** 2026-06-30

**Authors:** Jinwei Ma, Jiumao Cai, Meian Li, Lumeng Chao

**Affiliations:** 1 Computer and Information Engineering College, Inner Mongolia Autonomous Region Key Laboratory of Big Data Research and Application of Agriculture and Animal Husbandry, Inner Mongolia Agriculture University, Hohhot, China; 2 Institute of Farmland Irrigation of Chinese Academy of Agricultural Sciences, Xinxiang, China

**Keywords:** discrete wavelet transform, Kalman filtering, precision irrigation, soil moisture prediction, time series segmentation

## Abstract

Efficient soil moisture prediction is essential for improving precision irrigation practices, promoting sustainable water use, and mitigating crop water stress to enhance yields in irrigated farmlands. However, most prediction models rely on large dataset and high computational resources, limiting their applicability in resource-constrained agricultural environments. This study proposes a lightweight and cost-effective soil moisture prediction model tailored for precision irrigation applications. The method integrates discrete wavelet transform (DWT) and autocorrelation analysis to capture temporal dynamics in soil volumetric water content (VWC) time series. Specifically, DWT is employed to identify change points and segment the time series into irrigation-cycle-based subsequences, while autocorrelation features are extracted to characterize temporal dependencies. These features are subsequently incorporated into a Kalman filtering (KF) framework for recursive prediction. To improve adaptability under varying field conditions, a dynamic updating and rolling prediction mechanism based on a sliding window is introduced, allowing continuous incorporation of newly observed irrigation cycles and removal of outdated information. Results demonstrate that the proposed model achieves competitive prediction accuracy with substantially reduced computational cost. Its low complexity and adaptability make it well suited for real-time deployment on low-cost edge devices in precision irrigation systems.

## 1 Introduction

Agriculture accounts for nearly 70% of global freshwater consumption (Jalivand et al., 2019). Small-scale farms represent approximately 84% of all farms but contribute only 35% of global food production (Seo et al., 2025). Enhancing irrigation scheduling through intelligent technologies can improve crop productivity, reduce costs, and support the sustainable development of smallholder agriculture, thereby contributing to global food security (Gao et al., 2023).

Real-time decision-making for precision irrigation is a key component of smart irrigation systems. Existing irrigation decision-making approaches can be broadly categorized into four types: methods based on evapotranspiration and water balance, soil moisture measurements, plant growth indicators, and process-based agricultural models (Gu et al., 2020). The evapotranspiration and water balance-based method, which supplies water to crops based on their water requirements, is considered an ideal irrigation strategy. However, this method relies heavily on models and numerous meteorological parameters, which can lead to error accumulation and the need for more sensors, making it less favorable in terms of accuracy and cost (Abdelmoneim et al., 2025). Higher precision calculations require complex equipment and sensors, and the associated high costs and complexity hinder its widespread application (Jenkins and Block, 2024). The plant growth parameter-based method determines the water status by monitoring the plant’s physiological responses, providing an accurate reflection of the crop’s actual water needs. However, its application is limited by environmental sensitivity and the complexity of monitoring (Noun et al., 2022). Although process-based agricultural models offer rich agricultural process simulations, their applicability in variable environments is limited, especially when input data is insufficient. In such cases, the model’s results may be significantly biased, and the high demands for data quality and quantity require substantial computational and equipment investments (Tolomio and Casa, 2020; Mgana et al., 2026). Among these, soil moisture directly reflects the available water status for crops, offering advantages such as timely response, simplicity in measurement, and intuitive decision-making. It strikes an optimal balance between precision, cost, and operability in precision irrigation systems (Datta et al., 2023; Zhang et al., 2024; Zheng et al., 2026).

With the rapid development of the Internet of Things (IoT) and remote sensing technologies, soil moisture data acquisition has become increasingly refined, offering higher precision and spatiotemporal resolution. These data that provide a foundation for soil moisture prediction are primarily obtained through *in situ* sensors and satellite-based remote sensing systems (Loconsole et al., 2025). While each approach has its own advantages, soil moisture variability is influenced by multiple environmental factors, and single-point sensors have limited ability to capture spatial heterogeneity. This often necessitates the deployment of multiple sensors along with edge computing and wireless communication devices (Zhang et al., 2025). Remote sensing, on the other hand, requires substantial investment and generates large volumes of data, leading to increased storage and processing demands (Dorigo et al., 2017).

Recent studies have applied various artificial intelligence techniques to soil moisture prediction, including machine learning, deep learning, and emerging exploratory large language model-based approaches (Islam et al., 2025; Ivanova, 2025). Traditional machine learning models, such as decision trees, random forests, and XGBoost, are widely used due to their interpretability and computational efficiency (Togneri et al., 2022; Shan and Zheng, 2023; Teshome et al., 2024). Deep learning models, including artificial neural networks (ANN), convolutional neural networks (CNN), recurrent neural networks (RNN), and long short-term memory (LSTM) networks, have demonstrated strong capability in capturing temporal dependencies (Park et al., 2023; Datta and Faroughi, 2023; Li et al., 2022; Zhu et al., 2025). In particular, LSTM models are extensively used in time series prediction tasks. Hybrid models that integrate multiple architectures and attention mechanisms have further improved performance (Liu et al., 2024; Wang et al., 2024; Xu et al., 2024; Pan et al., 2025). Although the application of large language models in soil moisture prediction is still emerging, recent studies have highlighted their potential in improving both prediction accuracy and interpretability (Deforce et al., 2024; Koné et al., 2025).

Despite these advances, several limitations persist. First, most models require extensive long-term datasets, which are costly and impractical for smallholder farmers. Second, many models are designed for specific crops or environmental conditions, restricting their generalizability. Third, deep learning models often fail to explicitly capture the intrinsic temporal evolution patterns of soil moisture, which may consequently lead to overfitting and reduced interpretability.

To address these challenges, this study aims to develop a real-time adaptive soil moisture prediction model for irrigated farmlands under limited data and low-cost conditions. In this study, Discrete Wavelet Transform (DWT) is applied to decompose the soil moisture data into multiple irrigation subsequences. Each subsequence is represented using a linear model to characterize the average attenuation level and structure of the soil moisture. These linear models are then incorporated into the feature equations of the Kalman Filter (KF) algorithm, enabling the KF to track the soil moisture and estimate its value at the subsequent time step. In this approach, changes in soil moisture are treated as a state trajectory within the time series, thereby transforming the soil moisture prediction problem into a state trajectory estimation problem (Debnath et al., 2024; Li Y. et al., 2023).

The main contributions of this study are summarized as follows:

1. A time series segmentation and modeling framework for soil moisture data is proposed. Change points are detected using DWT, and the time series is segmented into homogeneous subsequences corresponding to irrigation cycles, thereby reducing modeling complexity.
2. A low-order autoregressive model is integrated into a Kalman filtering framework. Autocorrelation analysis of neighboring subsequences is used to construct a computationally efficient model, which is embedded into a state-space representation in Kalman filtering to improve prediction accuracy while maintaining efficiency.
3. A rolling update mechanism is designed to dynamically update subsequences, enabling the model to adapt to evolving environmental conditions. In addition, a multi-scale downsampling strategy is introduced to extend the prediction horizon.

**Figure 1:**
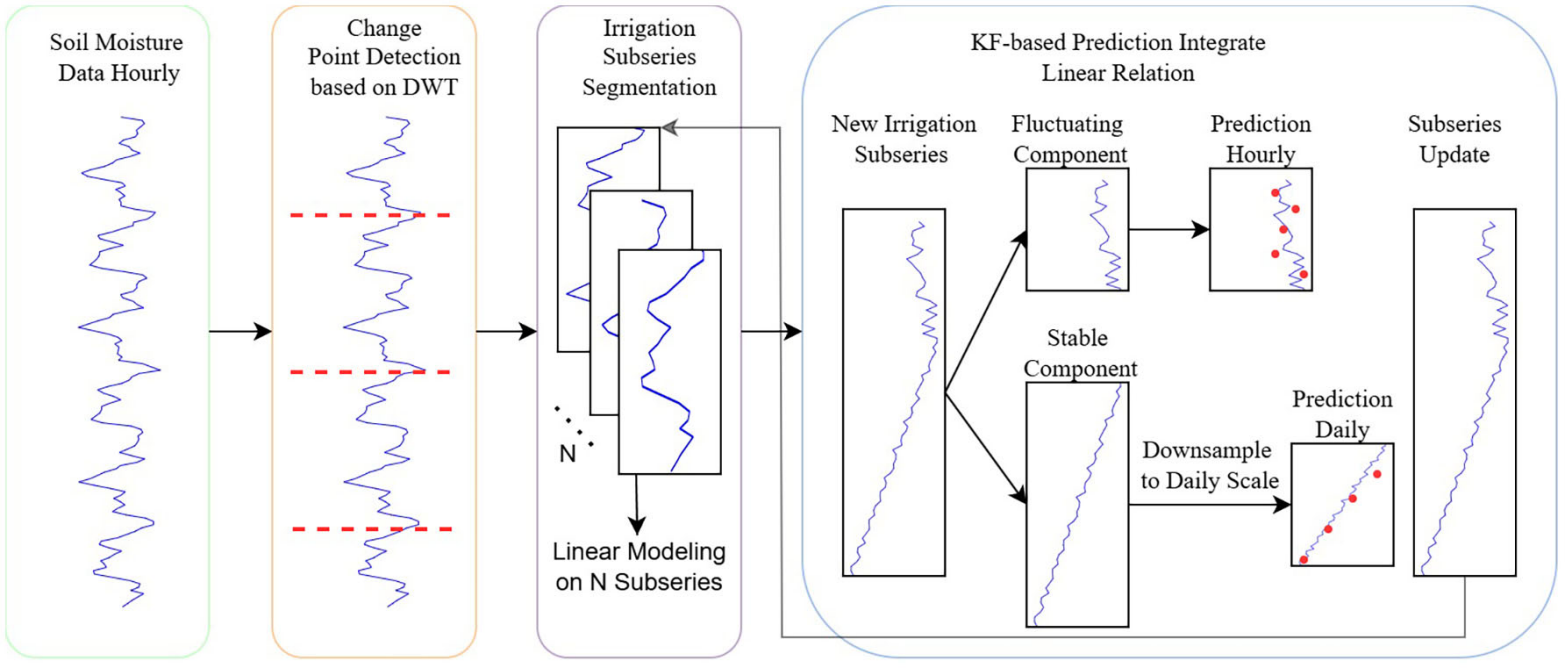
Technical flowchart of the soil moisture prediction framework proposed in this study.

**Figure 1** presents the detailed technical flowchart of the soil moisture prediction model proposed in this study.

## 2 Materials and methods

### 2.1 Problem formulation

Crop water uptake is governed by complex interactions among soil conditions, environmental factors, irrigation, and precipitation. To simplify this system, several quantitative indicators are commonly used, including soil moisture (SM), evapotranspiration (ET), and crop-related variables (Abioye et al., 2020).

**Figure 2:**
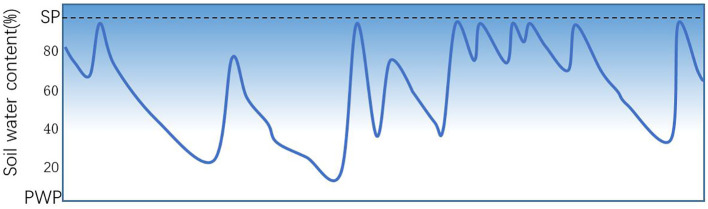
A common example of irrigated farmland soil moisture variation, where the soil water content values are between SP and PWP.

In irrigated agricultural systems, soil moisture typically fluctuates within bounded limits. The permanent wilting point (PWP) defines the lower bound, below which crops cannot extract water, while the saturation point (SP) represents the upper bound (Eisenhauer et al., 2021), as shown in **Figure 2**. In practice, field capacity (FC) and management allowable depletion (MAD) are often used as operational thresholds.

These constraints result in soil moisture time series exhibiting bounded fluctuations, where non-stationarity is primarily reflected in seasonal variations rather than long-term trends. Therefore, conventional statistical methods based solely on mean and variance may be insufficient to adequately capture non-stationary characteristics (Ye et al., 2024).

In contrast, frequency-domain methods, such as Fourier and wavelet transforms, are effective in identifying periodic structures and detecting change points (Ghaderpour, 2021). Wavelet transform, in particular, provides strong time–frequency localization, enabling the identification of transient features at multiple resolutions (Mallat, 2009).

Based on these properties, wavelet-based adaptive change point detection is employed to segment soil moisture time series data into semantically meaningful subsequences (Li et al., 2023). These subsequences correspond to irrigation cycles, during which soil functions as a storage unit: water enters through irrigation or precipitation and gradually decreases due to evaporation, percolation, and plant uptake (Allen et al., 1998; Novák, 2012).

Accordingly, the prediction task is reformulated to estimate soil moisture dynamics within individual irrigation cycles, with a particular emphasis on the depletion phase, in order to inform irrigation management strategies that mitigate crop yield losses caused by water stress (Zhang et al., 2020).

### 2.2 Proposed method

#### 2.2.1 Model architecture

**Figure 3:**
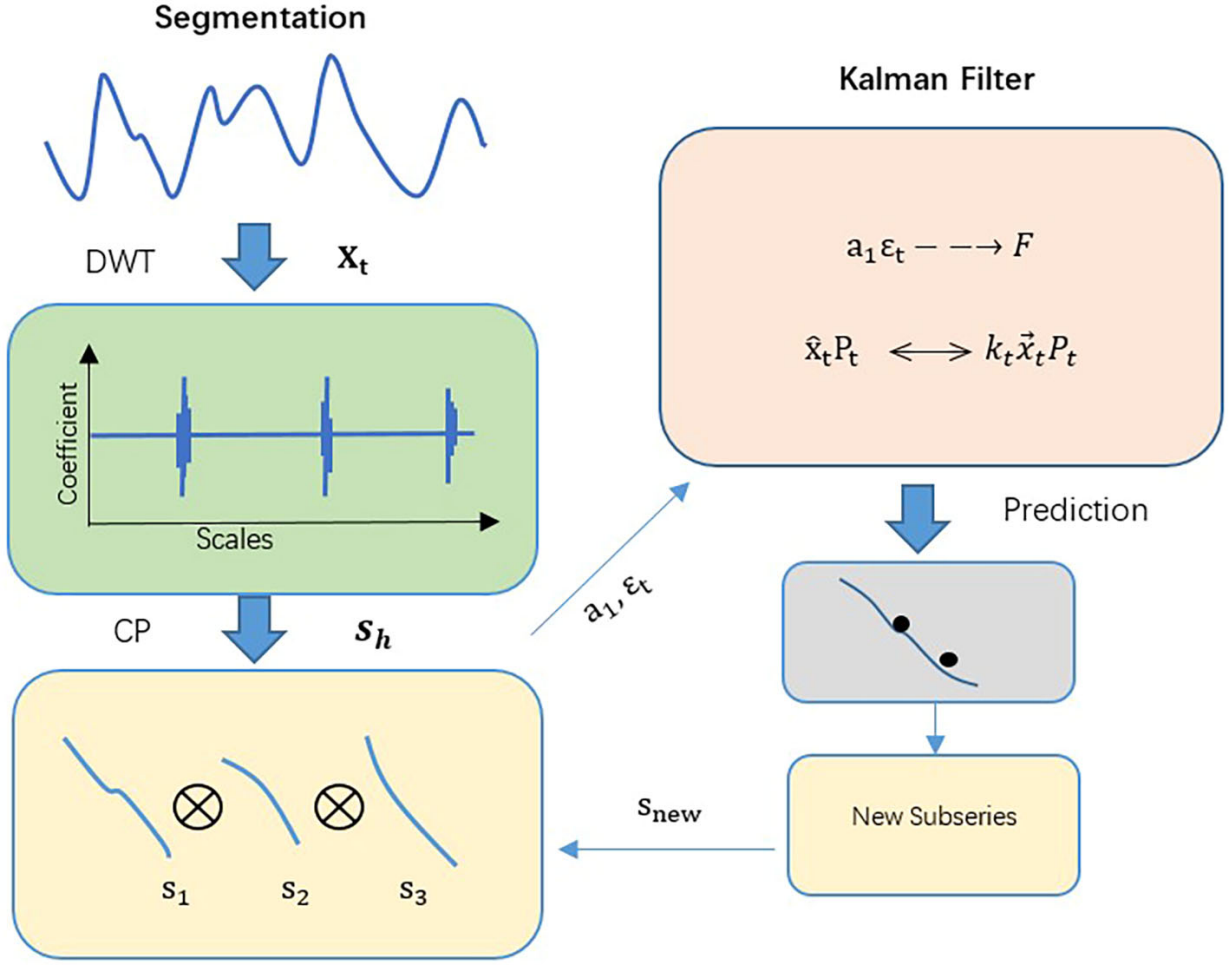
An overview of the proposed method which consists of time series segmentation, subsequence analysis and Kalman filter prediction.

Given a univariate soil moisture time series $\text{X}\in R^{N\times 1}$ for an irrigated farmland, where N is the total number of time steps, multiple effective change points are detected using the DWT. Among these, H change points closest to the prediction target are selected to construct a sliding window, dividing the original sequence into H subsequences $S_{h}$, forming the subsequence set S. For each subsequence $S_{h}$, a first-order autoregressive model (AR(1)) is applied to estimate the autoregressive coefficient $a_{h1}$ and the residuals $\;\epsilon_{h}$. Then, a linear weighting scheme is used within the sliding window to obtain the final regression coefficient $a_{1}$ and residual ϵ. At this point, the prediction task can be expressed as: $\;x_{t}=a_{1}x_{t-1}+\epsilon$. This formulation approximates soil moisture dynamics as a linear process within each irrigation cycle. However, such simplification may reduce model accuracy. To address this limitation, the linear model is incorporated into the state transition equation F of the Kalman filtering. By analyzing the residuals, the process noise covariance Q is adaptively adjusted. Furthermore, as new irrigation subsequences emerge, the subsequence set *S* is dynamically updated, enabling adaptive variation of the autoregressive model and rolling prediction within the Kalman filtering framework. The overall computational complexity of the proposed method is low, making it suitable for real-time applications on resource-constrained devices. The overall model architecture is illustrated in **Figure 3**.

#### 2.2.2 Soil moisture time series segmentation

In this study, the DWT is employed for segmenting soil moisture time series data. DWT is well suited for analyzing non-stationary signals, as it decomposes the data into multiple frequency components corresponding to different temporal scales. This enables the detection of change points associated with significant transitions in soil moisture levels, which are critical for irrigation management. Once change points are identified, the time series is divided into multiple homogeneous subsequences, each representing a distinct irrigation cycle or phase of soil moisture evolution. This segmentation reduces the complexity of modeling long time series by focusing on shorter periods with similar patterns, thereby improving prediction accuracy.

High-frequency detail coefficients are extracted from the time series using DWT. Change points are then identified by applying a threshold to select coefficients with large magnitudes, thereby achieving time series segmentation. Wavelet transform is a time frequency analysis method that decomposes a signal using a set of wavelet basis functions. This decomposition produces approximation and detail coefficients at different scales and positions, capturing local variations in both time and frequency domains. In particular, detail coefficients highlight abrupt changes, transients, and localized features in the signal. The continuous wavelet transform (CWT) of a signal x(t) is defined as (Debnath and Shah, 2015):


$$W_{x}(s,\tau )=\frac{1}{\sqrt{s}}\int_{-\infty }^{\infty }x(t)\psi_{s,\tau }^{*}(t)dt$$

where ψ(t) denotes the mother wavelet function and $\psi^{*}(t)$ denotes the complex conjugate. The parameter s is the scale factor controlling the frequency resolution, and τ is the translation parameter determining the temporal location of the analysis. The coefficient $\;W_{x}(s,\tau )$ characterizes the similarity between the signal and the wavelet function at a specific scale and time.

The DWT represents a discrete time series as a set of wavelet coefficients derived from the CWT. These coefficients are obtained using a pair of high-pass and low-pass filters (Sundararajan, 2016). The low-pass filter h produces approximation coefficients, while the high-pass filter g extracts detail coefficients. The decomposition equation is given by:


$$a_{i}[k]=\sum_{p}x[p]\cdot h[2k-p]$$

$$d_{i}[k]=\sum_{p}x[p]\cdot g[2k-p]$$

$a_{i}[k]$ represents the approximation coefficients, while $d_{i}[k]$represents the detail coefficients.

In this study, the Daubechies 4 (db4) wavelet is selected, which is a widely used member of the Daubechies orthogonal wavelet family. It effectively captures both local features and long-term trends, making it suitable for change point detection and signal decomposition (Bolzan et al., 2020). The detected change points are then used to construct the subsequence set S, representing irrigation-cycle-based segmentation of the time series.

#### 2.2.3 Subsequence autoregressive analysis

In this study, each subsequence is modeled using AR(1), based on the analysis of the autocorrelation function (ACF) and partial autocorrelation function (PACF) (Hassani et al., 2024). The correlation analysis results are presented in the subsequent experimental section. Autoregressive models are widely used in time series analysis to describe the relationship between the current value and its previous observations. The general form of an autoregressive model is given by:


$$x(t)=\sum_{i=1}^{n}a_{i}x(t-i)+\epsilon (t)$$

where x(t)​ is the soil moisture value at time t, $a_{i}$​ is the autoregressive coefficient, and ϵ(t) represents the residual term.

For each segmented subsequence $S_{h}$, an AR(1) model is fitted, yielding the coefficient and residual $\left(a_{h1},\epsilon_{h}\right)$. To integrate information from multiple subsequences, a linear weighting scheme is applied within a sliding window of size H. The weight for each subsequence is defined as:


$$w_{h}=\frac{h}{\sum_{h=1}^{H}h}$$

where h​ is the index of the subsequence, starting from 1 indicating the farthest away from the prediciton target. The final autoregressive coefficient and residual are computed as weighted sum. The $a_{1}$ is defined as:


$$a_{1}=\sum_{h=1}^{H}w_{h}a_{h}$$

The ϵ is given by:


$$\epsilon =\sum_{h=1}^{H}w_{h}\epsilon_{h}$$

This formulation represents soil moisture dynamics as a weighted linear combination of autocorrelated processes, significantly reducing model complexity and computational cost. However, such simplification may lead to a loss of prediction accuracy. To mitigate this limitation, the model is further enhanced by incorporating a Kalman filtering framework with rolling prediction.

#### 2.2.4 Kalman filter prediction

The Kalman filtering is a recursive estimation algorithm widely used for state prediction and noise reduction in dynamic systems (Bai et al., 2023). It is particularly suitable for soil moisture prediction, as it can adapt to time-varying system dynamics (Gao et al., 2025). The Kalman filtering consists of two main steps: prediction and update.

In the prediction step, the system state is predicted based on the previous estimate and a state transition model:


$${\hat{x}}_{k}^{-}=F{\hat{x}}_{k-1}^{+}+Bu_{k}$$

$$P_{k}^{-}=FP_{k-1}^{+}F^{T}+Q$$

where $\hat{\text{x}}$ denotes the estimated state, F is the state transition matrix, B is the control matrix and P is the error covariance matrix, Q represents the process noise covariance matrix. The superscripts “ −” and “ +” denote the *a priori* and a posteriori estimates, respectively, and k indicates the time step. In this study, the autoregressive parameters (a1,ϵ) are incorporated into the state transition matrix F. Since the model considers a univariate system without external inputs, the control matrix is set to B=0.

In the update step, when a new observation becomes available, the state estimate is updated as follows:


$$K_{k}=P_{k}^{-}H^{T}{\left(HP_{k}^{-}H^{T}+R\right)}^{-1}$$

$${\hat{x}}_{k}^{+}={\hat{x}}_{k}^{-}+K_{k}(z_{k}-H{\hat{x}}_{k}^{-})$$

$$P_{k}^{+}=(I-K_{k}H)P_{k}^{-}$$

where K is the Kalman gain, H is the measurement matrix, R is the measurement noise covariance matrix, z is the measurement vector, and I is the identity matrix. The measurement residual is defined as:


$$y=z_{k}-H{\hat{x}}_{k}^{-}$$

This residual reflects the discrepancy between predicted and observed values and can be used to evaluate prediction uncertainty. When the residual exceeds a predefined threshold, the process noise covariance Q is increased adaptively to improve model responsiveness. The residual threshold in the Kalman filter was set to 0.5, based on the observation that residuals in stable soil moisture regions did not exceed this value. The observation noise covariance R was initialized to 1, and the process noise covariance Q was set such that Q/R = 1. When the residual exceeded the threshold, Q was increased tenfold, allowing rapid response to abrupt changes while maintaining smooth estimation in stable periods.

The KF is generally effective for short-term prediction at the original temporal resolution. However, such short horizons may have limited practical value for irrigation scheduling. To extend the prediction horizon, a multi-scale downsampling strategy is adopted.

Specifically, the original hourly time series is aggregated to coarser daily temporal scales according to the desired prediction horizon. The resulting time series is then segmented into irrigation-cycle subsequences, and the proposed modeling framework is applied. For example, a 1-day prediction is based on daily data, while longer horizons (e.g., 3–5 days) are achieved through further temporal aggregation. In this study, the maximum prediction horizon is set to 5 days. Longer horizons may span multiple subsequences, reducing prediction reliability.

Soil moisture evolves continuously over time, and new irrigation cycles emerge dynamically under changing environmental conditions, including variations in crop growth stages, radiation, and temperature.

To address these dynamics, a rolling update prediction mechanism is introduced. The model is constructed using the most recent h subsequences within a sliding window. As new subsequences become available, outdated ones are removed, ensuring that the model is continuously updated based on the latest data. This strategy enables the model to adapt to evolving environmental conditions and maintain prediction accuracy over time.

## 3 Results and discussion

### 3.1 Data source

**Figure 4:**
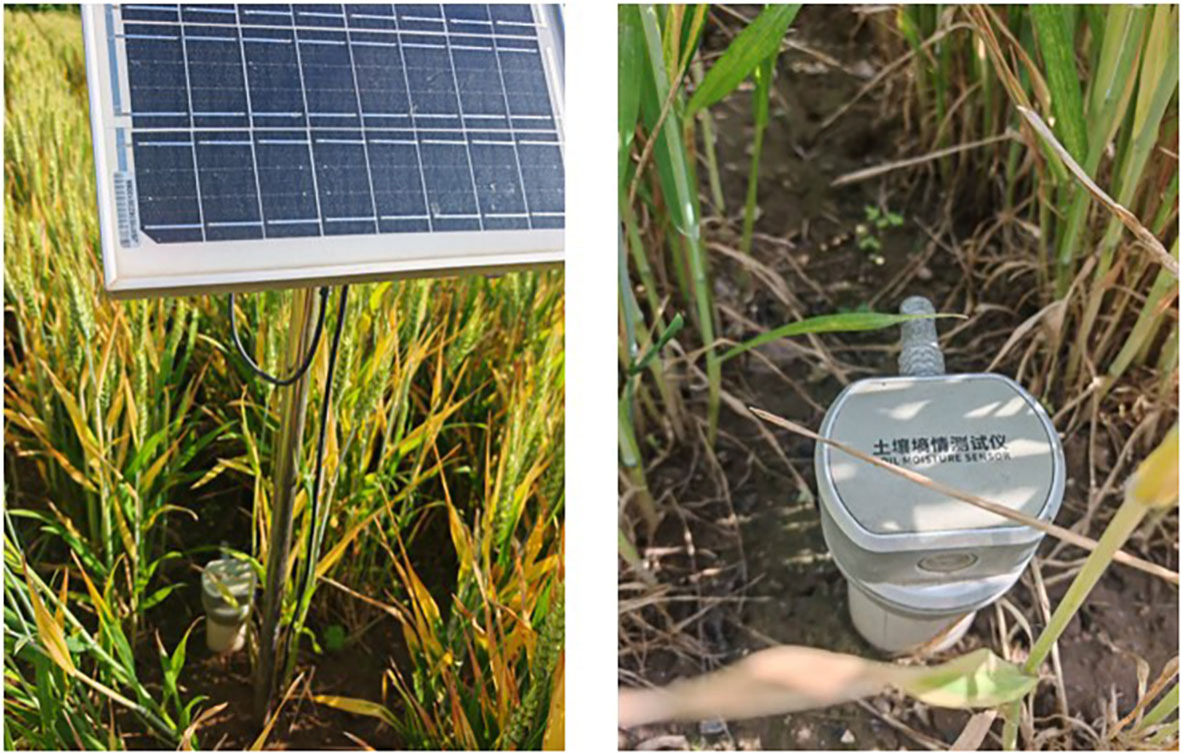
Soil moisture sensors and solar panels in the field.

The proposed algorithm was validated using field measurements collected from an irrigated farmland experimental site operated by the Institute of Farmland Irrigation of Chinese Academy of Agricultural Sciences in Xinxiang, Henan, China. The experimental site is located at an elevation of 80 m, with an annual mean temperature of 14.1 °C and an average annual precipitation of 582 mm. The groundwater table is at an average depth of 25 m, varying within ±2 m over the year. The soil consists of sandy loam and silty loam, and the influence of groundwater on root-zone soil moisture is negligible. The experimental farmland is cultivated with winter wheat and maize, following a winter wheat–maize rotation system. **Figure 4** illustrates the field deployment of the soil moisture sensing equipment.

The dataset was collected as part of a broader research project aimed at improving irrigation scheduling through integrated analysis of ecological and environmental information. Multiple sensors were deployed to monitor soil moisture, soil temperature, precipitation, and ambient temperature. These observations were further used to estimate evapotranspiration, crop water consumption, root-zone depth, and soil water balance for irrigation management.

Soil volumetric water content (VWC) and soil temperature data have been collected since March 2025 at depths ranging from 10 cm to 100 cm, with intervals of 10 cm, resulting in ten monitoring layers. In this study, the analysis focuses on soil moisture at depths of 20 cm and 30 cm, which are representative of the active root zone.

**Figure 5:**
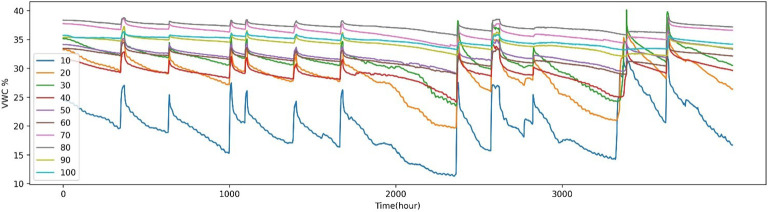
Hourly time series data of soil volumetric water content at all 10 different depths.

**Figure 5** shows the hourly soil moisture time series across all depth layers from 5 March 2025 to 20 August 2025. The subsequent validation is conducted using data from the 20 cm and 30 cm layers.

### 3.2 DWT-based time series segmentation and validation

A four-level wavelet decomposition was applied, and the detail coefficients at intermediate scales were selected for analysis. A four-level wavelet decomposition ensures the presence of intermediate detail components while maintaining minimal computational cost. Statistical analysis shows that the standard deviation of these coefficients is larger than the mean, which in turn exceeds the median, indicating a skewed distribution dominated by a small number of large coefficients.

Based on this observation, change points were identified by selecting coefficients exceeding the 95th percentile in absolute value. To improve robustness, neighboring change points within a short temporal interval were merged to eliminate redundant detections, resulting in a refined set of effective change points. We apply the k-means algorithm to merge adjacent change points and use the silhouette coefficient to determine the optimal value of k. The regions for change point merging correspond to high-frequency fluctuation areas, with durations reaching up to 56 hours. The first change point within each region is selected as the segmentation point, and a 2-day period is set to cover the high-frequency fluctuation area.

**Figure 6:**
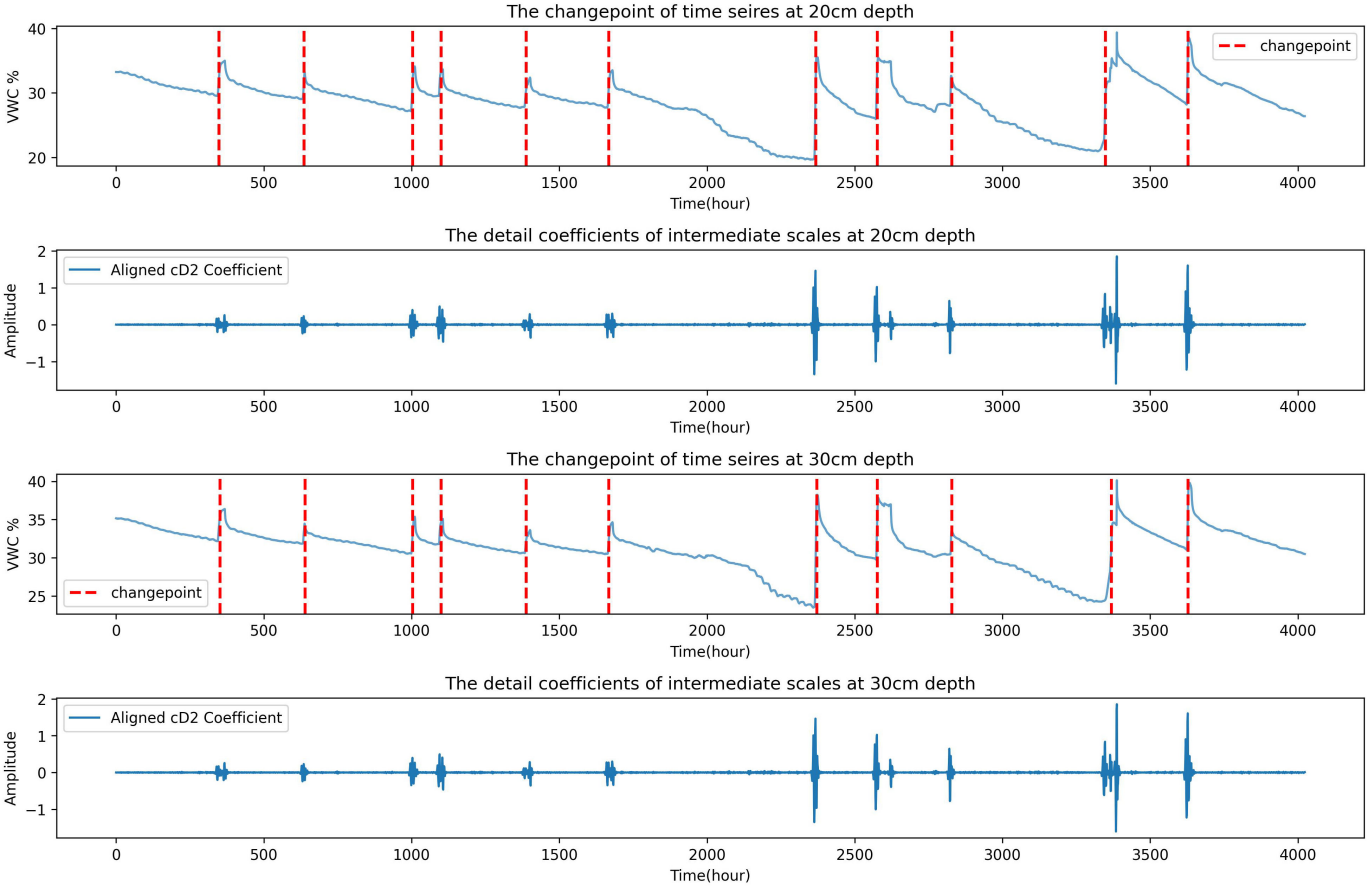
Detected change points in soil moisture time series at depths of 20 cm and 30 cm, and the detail coefficients at intermediate scales of discrete wavelet transform.

**Figure 6** illustrates the detected change points in soil moisture time series at depths of 20 cm and 30 cm. These points correspond to rapid increases in soil moisture, primarily caused by irrigation or precipitation events compared with irrigation and rainfall records. During such events, water rapidly infiltrates soil pores, displacing air and leading to a sharp increase in volumetric water content (Cui et al., 2025).

In contrast, soil moisture depletion occurs more gradually through evaporation, percolation, and plant uptake. Additionally, soil water retention properties further slow down the depletion process, resulting in a nonlinear decay pattern (Merk et al., 2021).

Based on the detected change points, the time series is segmented into multiple irrigation-cycle subsequences, each representing a distinct soil moisture evolution phase following irrigation or rainfall events.

To support multi-day prediction, the original hourly data are downsampled to a daily scale. The detected change points are then mapped to the downsampled series for segmentation. However, for certain irrigation scenarios, subsequence downsampling is not always necessary. For instance, in high-frequency short-term irrigation scenarios, the irrigation cycle may be shorter than 2 days, where the prediction horizon is no longer on a daily scale but instead focuses on soil moisture at 1-hour, 3-hour, or 5-hour intervals. Different irrigation scenarios involve varying irrigation cycle scales, and accordingly, the prediction horizon should be adjusted. Whether or not to downsample the data depends on the scale of the prediction horizon.

Historical subsequences shorter than 5 days are discarded in autoregressive model analysis. These short subsequences require further downsampling in 3-day or 5-day predictions to establish linear relationships. However, after downsampling, the number of observation points may fall below 3, making it impossible to build a linear model.

The daily-level prediction for the entire irrigation subsequence primarily focuses on the soil moisture attenuation process, excluding the high-frequency fluctuation areas caused by irrigation during the first two days of the subsequence. Fluctuation areas are monitored using sensors, with hourly-level predictions employed to assess the effectiveness of irrigation implementation.

**Figure 7:**
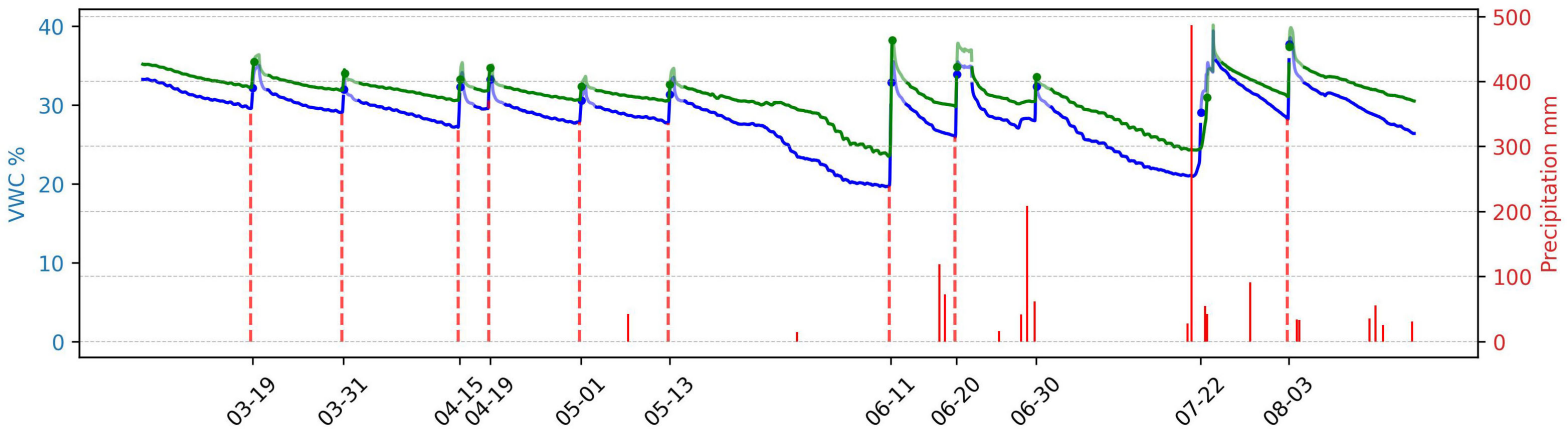
Hourly segmented subsequences results, validated against irrigation and precipitation events. In this graph, blue represents soil moisture at 20 cm depth, and green represents 30 cm depth. In the blue and green areas, the light semi-transparent shading represents stable regions, while the dark opaque shading denotes fluctuating regions over two days. The left y-axis corresponds to soil moisture values. Circles denote detected change points, with the x-axis showing the timing of the change points (only 20 cm depth is shown, as the two depths exhibit similar change points). Red dashed lines indicate irrigation events, while solid red lines represent precipitation events and precipitation amounts. The right y-axis corresponds to precipitation values.

Hourly soil moisture time series at depths of 20 cm and 30 cm were segmented, and the results were validated against irrigation and precipitation events. The resulting subsequences are presented in **Figure 7**.

As shown in **Figure 7**, the change points detected using DWT align well with irrigation and precipitation events. In July, two irrigation cycles correspond to precipitation events with relatively substantial rainfall. The time lag between irrigation events and the detected change points is approximately 3 hours, whereas the lag between precipitation events and change points is longer and exhibits no clear pattern. The two-day duration generally encompasses the periods of high soil moisture variability induced by irrigation and precipitation. These observations validate the effectiveness of the proposed irrigation-cycle-based segmentation method for soil moisture time series data.

### 3.3 Subsequence data analysis

The identification of the time series model was based on the autocorrelation function (ACF), partial autocorrelation function (PACF), and the Akaike Information Criterion (AIC). For each subsequence, a first-order autoregressive model (AR(1)) was selected.

**Figure 8:**
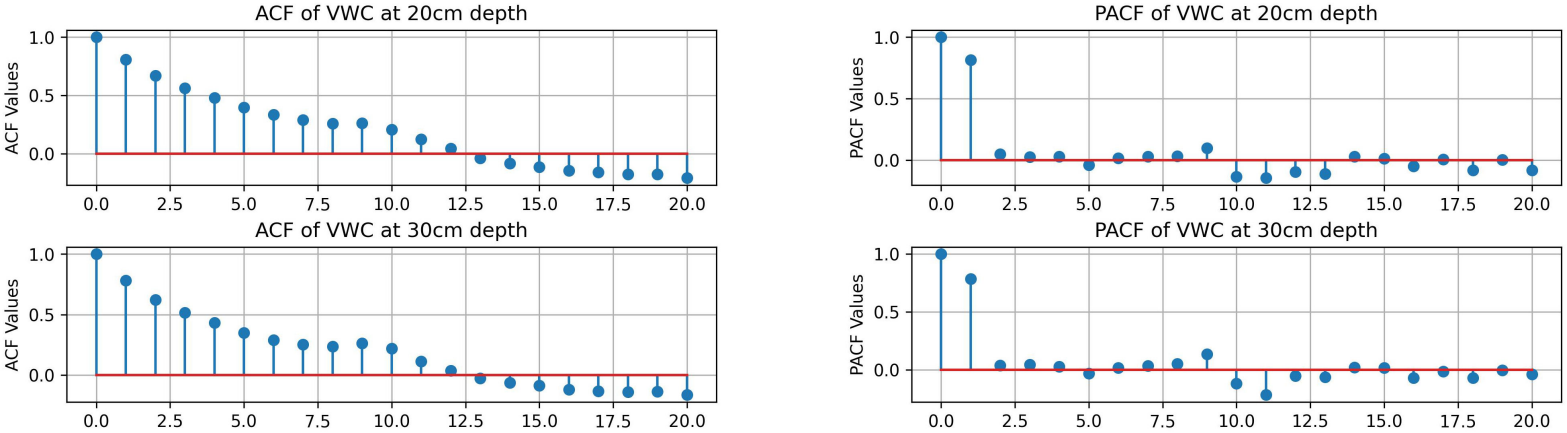
Autocorrelation analysis based on ACF and PACF.

As shown in **Figure 8**, the ACF exhibits a gradual decay (long tail), while the PACF shows a sharp cutoff after the first lag. These characteristics indicate that an AR(1) model is appropriate for representing the underlying temporal dependence structure.

Each subsequence is fitted using the AR(1) model to obtain the corresponding coefficients, which are then aggregated to derive the final model parameters. This linear relationship describes the average process and structure of soil moisture attenuation within the irrigation cycle. As fundamental prior knowledge and mechanisms, it enhances the stability and noise robustness of the Kalman filter algorithm in small sample scenarios, while also offering interpretability in practical applications (Wang et al., 2023).

The sliding window size is set to h=6. Due to the irregular and variable length of irrigation cycles, longer cycles can capture more information related to irrigation patterns. In addition, different soil types exhibit varying soil moisture memory times (Martínez-Fernández et al., 2020; Rahman et al., 2015), with clay soils having the longest memory, reaching up to 3–6 irrigation cycles. Therefore, the maximum number of irrigation cycles, 6 is selected as the sliding window size.

To ensure model stability, a constraint is imposed such that the autoregressive coefficient satisfies $|{\text{a}}_{h1}|<1$. This prevents divergence of predictions and excessive error accumulation as the prediction horizon increases. Based on the experimental data presented in this study, for the soil moisture attenuation process, the influence of soil moisture at a given time on subsequent moisture values gradually diminishes over time. This coefficient can be interpreted as the statistical persistence of soil moisture within a subsequences, and no negative coefficients were observed.

### 3.4 Model prediction performance

**Figure 9:**
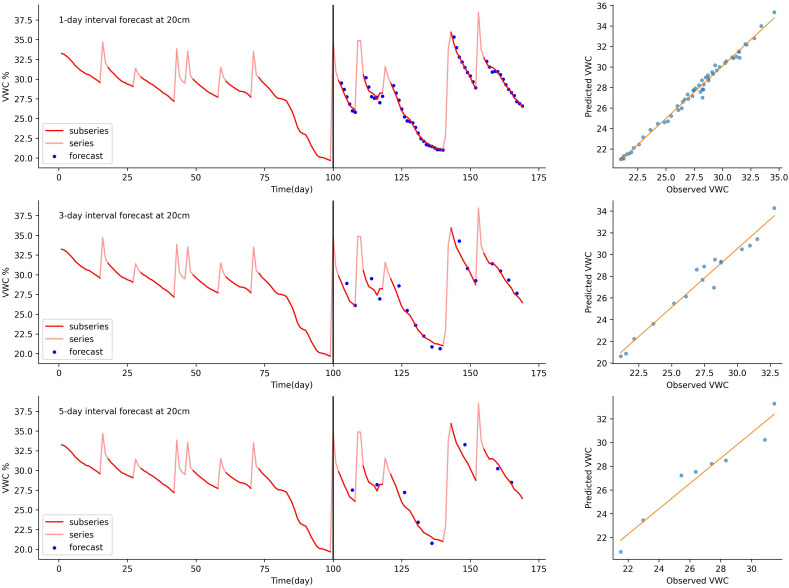
Left column: Soil moisture prediction results at 20 cm depth for 1-day, 3-day, and 5-day prediction horizons. The actual observations are divided into left and right parts by a vertical line(Time = 100 days), with the left used for subsequence correlation analysis and the right for prediction validation. Right column: The discrepancy between the observed and predicted values of VWC, with the solid line indicating the perfect agreement.

**Figure 10:**
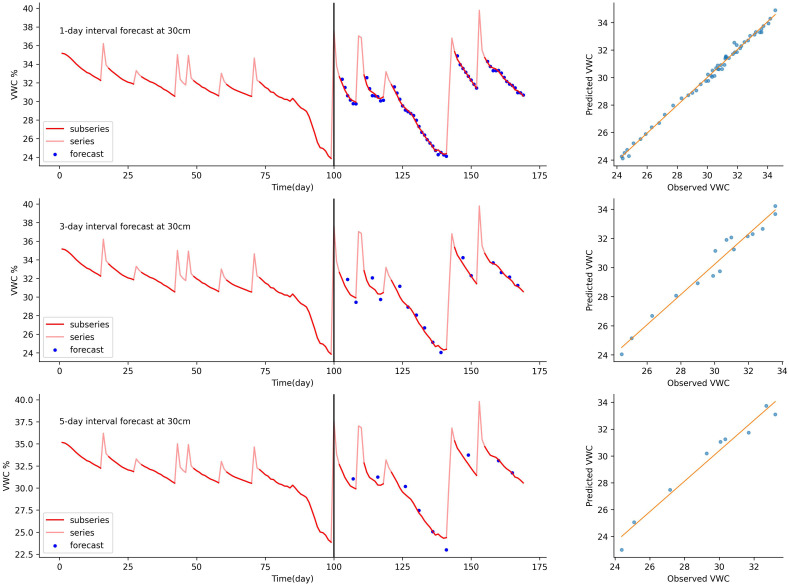
Left column: Soil moisture prediction results at 30 cm depth for 1-day, 3-day, and 5-day prediction horizons. The actual observations are divided into left and right parts by a vertical line(Time = 100 days), with the left used for subsequence correlation analysis and the right for prediction validation. Right column: The discrepancy between the observed and predicted values of VWC, with the solid line indicating the perfect agreement.

**Figure 9** and **10** present the soil moisture prediction results at depths of 20 cm and 30 cm for prediction horizons of 1, 3, and 5 days. In these experiments, the first six subsequences were used for model construction, while the subsequent five subsequences were used for validation. Predictions were performed in a one-step-ahead manner based on observed values from the previous time step.

In **Figure 9**, for the 20 cm depth, the coefficient of determination ${\text{R}}^{2}$ values were 0.9896, 0.9394, and 0.8883 for the 1-day, 3-day, and 5-day horizons, respectively. The mean absolute percentage error (MAPE) values were 0.0098, 0.0225, and 0.0351, while the root mean square error (RMSE) values were 0.0035, 0.0085, and 0.0109 ${\text{m}}^{3}/{\text{m}}^{3}$.

In **Figure 10**, for the 30 cm depth, similar trends were observed. The ${\text{R}}^{2}$ values were 0.9940, 0.9552, and 0.9288, while the MAPE values were 0.0054, 0.0146, and 0.0223, and the RMSE values were 0.0022, 0.0058, and 0.0080 ${\text{m}}^{3}/{\text{m}}^{3}$.

Overall, the results indicate that the proposed model effectively captures soil moisture dynamics at both depths, achieving high accuracy for short-term predictions. As the prediction horizon increases, the accuracy gradually decreases. This decline can be attributed to error accumulation and the reduced effectiveness of Kalman filtering updates over longer prediction intervals. These results indicate that the proposed model is particularly suitable for short-term irrigation decision-making, where high accuracy and rapid response are required.

### 3.5 Rolling update prediction

The previous experiments were conducted without incorporating the rolling update mechanism. In this section, rolling prediction is applied to soil moisture data after day 100. Newly detected subsequences are continuously incorporated into the sliding window, replacing outdated ones.

**Figure 11:**
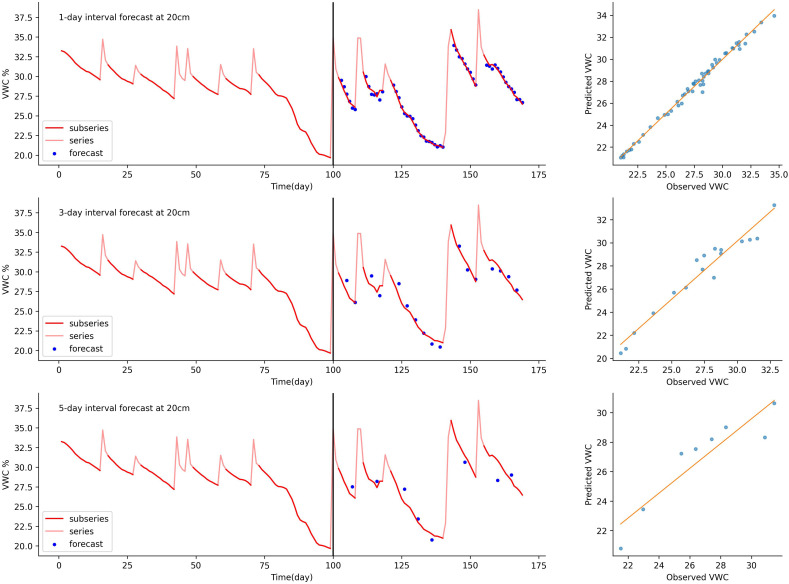
Left column: Soil moisture prediction results at 20 cm depth for 1-day, 3-day, and 5-day prediction horizons with subsequence update rolling prediction. The actual observations are divided into left and right parts by a vertical line(Time = 100 days), with the left used for initial subsequence correlation analysis and the right for prediction validation. Right column: The discrepancy between the observed and predicted values of VWC, with the solid line indicating the perfect agreement.

In **Figure 11**, for the 20 cm depth, the ${\text{R}}^{2}$ values were 0.9912, 0.9408, and 0.8431 for the 1-day, 3-day, and 5-day horizons, respectively. The corresponding MAPE values were 0.0090, 0.0249, and 0.0412, while the RMSE values were 0.0033, 0.0082, and 0.0130 ${\text{m}}^{3}/{\text{m}}^{3}$.

**Figure 12:**
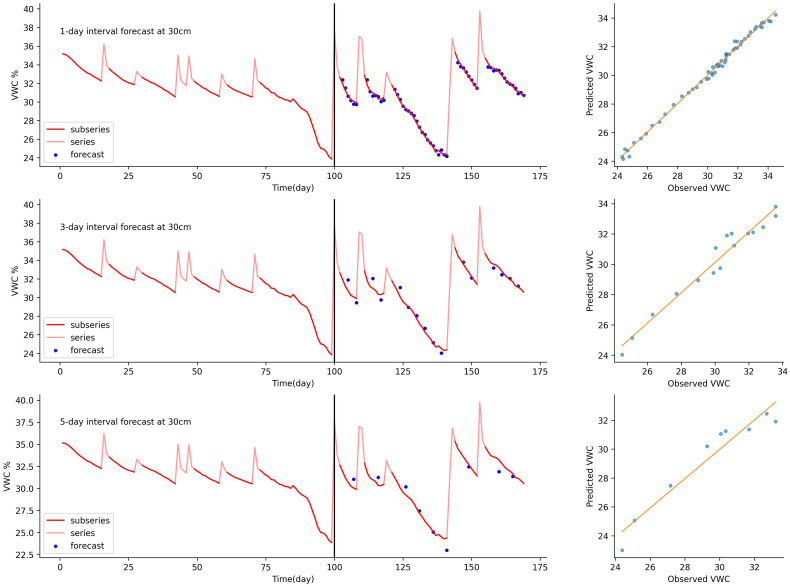
Soil moisture prediction results at 30 cm depth for 1-day, 3-day, and 5-day prediction horizons with subsequence update rolling prediction. The actual observations are divided into left and right parts by a vertical line(Time = 100 days), with the left used for initial subsequence correlation analysis and the right for prediction validation. Right column: The discrepancy between the observed and predicted values of VWC, with the solid line indicating the perfect agreement.

In **Figure 12**, for the 30 cm depth, the ${\text{R}}^{2}$ values were 0.9945, 0.9578, and 0.9189, while the MAPE values were 0.0055, 0.0145, and 0.0246, and the RMSE values were 0.0020, 0.0056, and 0.0085 ${\text{m}}^{3}/{\text{m}}^{3}$. **Table 1** presents a comparison of these prediction results.

**Table 1:** A comparison of the 1-day, 3-day, and 5-day single-step soil moisture prediction results at soil depths of 20 cm and 30 cm is presented, using both rolling and non-rolling prediction methods. The unit of RMSE is m³/m³.

Soil depth	Prediction horizon	Rolling prediction			Model prediction		
Depth	Horizon	${\text{R}}^{2}$	RMSE	MAPE	${\text{R}}^{2}$	RMSE	MAPE
20cm	1day	0.9912	0.0033	0.0090	0.9896	0.0035	0.0098
	3day	0.9408	0.0082	0.0249	0.9394	0.0083	0.0225
	5day	0.8431	0.0130	0.0412	0.8883	0.0109	0.0351
30cm	1day	0.9945	0.0021	0.0055	0.9940	0.0022	0.0054
	3day	0.9579	0.0056	0.0145	0.9552	0.0058	0.0146
	5day	0.9189	0.0085	0.0246	0.9288	0.0080	0.0223

The results show that rolling updates improve prediction accuracy for short-term horizons (1–3 days) by incorporating the most recent system dynamics. However, for the 5-day horizon, performance decreases slightly. The decline in prediction performance can be attributed to the attenuation or omission of subsequences that contain richer long-term dependencies during the updating process. This indicate that the simple time-based rolling strategy used for subsequence updating still has room for improvement. The 5-day single-step prediction results at a depth of 30 cm outperform those at 20 cm under both prediction approaches, indicating that deeper soil layers are generally less affected by surface evaporation and exhibit lower sensitivity to short-term atmospheric fluctuations, thereby showing more stable soil moisture dynamics.

### 3.6 Prediction model performance comparison

This section compares the proposed model with representative machine learning and deep learning approaches in terms of prediction accuracy and model complexity.

Machine learning methods, such as LightGBM, rely on feature engineering, while deep learning models, such as LSTM, require large datasets for effective training. Although these approaches demonstrate strong capability in capturing nonlinear relationships, they typically involve higher computational costs.

In contrast, the proposed model operates in a lightweight and real-time manner, relying on historical irrigation cycle patterns. It requires fewer data and has significantly lower computational complexity, making it suitable for deployment on edge devices.

After analysis of model complexity, an important question arises: how much accuracy can be achieved using simpler models? To address this, LightGBM is selected as a representative machine learning model due to its strong performance among ensemble learning methods. For deep learning, the LSTM model is chosen as a representative approach widely used in time series prediction tasks.

Due to the limited size of the downsampled daily dataset, LightGBM and LSTM are unable to effectively train reliable models. Therefore, the performance comparison is conducted using hourly data. **Table 2** presents a comparison of the rolling Kalman filter (KF) algorithm with LightGBM and LSTM in predicting soil moisture at horizons of 1 h, 3 h, and 5 h. These predictions capture the full dynamics of soil moisture, including both fluctuating and steady trend components. For both LSTM and LightGBM, 70% of the dataset is used for training and 30% for testing. The models utilize the previous 7 soil moisture observations to perform single-step soil moisture prediction.

**Table 2:** A comparative analysis of hourly soil moisture prediction performance at depths of 20 cm and 30 cm is conducted for prediction horizons of 1 h, 3 h, and 5 h, using rolling Kalman filter (KF) forecasting, LSTM, and LightGBM methods. The unit of RMSE is m³/m³.

Soil depth	Prediction horizon	KF (Rolling prediction)			LSTM			LightGBM		
Depth	Horizon	${\text{R}}^{2}$	RMSE	MAPE	${\text{R}}^{2}$	RMSE	MAPE	${\text{R}}^{2}$	RMSE	MAPE
20cm	1hour	0.9948	0.0027	0.0019	0.9807	0.0058	0.0063	0.9895	0.0043	0.0049
	3hour	0.9826	0.0041	0.0037	0.9650	0.0079	0.0084	0.9734	0.0069	0.0089
	5hour	0.9787	0.0054	0.0066	0.9401	0.0103	0.0108	0.9520	0.0092	0.0121
30cm	1hour	0.9951	0.0022	0.0013	0.9868	0.0039	0.0036	0.9907	0.0032	0.0036
	3hour	0.9884	0.0033	0.0030	0.9711	0.0057	0.0056	0.9755	0.0053	0.0056
	5hour	0.9743	0.0048	0.0051	0.9469	0.0078	0.0069	0.9530	0.0073	0.0084

The comparative results of the prediction performance experiments demonstrate that the lightweight prediction model proposed in this study outperforms LSTM and LightGBM overall. It does not require large amounts of data for training and is still capable of performing prediction tasks effectively on limited downsampled daily data. Moreover, it can flexibly adapt the prediction scale according to different scenarios, achieving high accuracy while exhibiting improved adaptability and robustness.

The computational efficiency of these models is summarized in **Table 3**, which compares training data requirements, inference time, and parameter counts.

**Table 3:** A comparative analysis of hourly computational efficiency performance using rolling Kalman filter (KF) forecasting, LSTM, and LightGBM methods.

Model	Training time(s)	Inference time(s)	Parameters
KF+Rolling	0	0.6170	14
LSTM	13.79	0.0020	379
LightGBM	0.21	0.0014	9000

Although LightGBM shows slightly lower overall training and inference times, the proposed method is more suitable for deployment on resource-constrained edge devices in precision irrigation systems. This advantage is due to its minimal parameter count, extremely low memory usage, negligible training data requirements, and support for real-time rolling prediction.

A key advantage of the proposed model lies in its adaptability and interpretability. By segmenting soil moisture dynamics into irrigation-cycle-based subsequences, the model effectively reduces the impact of non-stationarity while preserving the underlying physical patterns.

Furthermore, the integration of a rolling update mechanism enables the model to continuously adapt to changing environmental conditions, such as variations in climate and crop growth stages. This adaptability is particularly important for real-world agricultural applications, where system dynamics are inherently time-varying.

Overall, the proposed model achieves a favorable balance between prediction accuracy and computational efficiency, making it a practical solution for real-time soil moisture prediction in resource-constrained agricultural systems.

### 3.7 Limitations and future work

Although the proposed model demonstrates strong performance in soil moisture prediction, several limitations should be acknowledged.

First, the model relies on change points detected via Discrete Wavelet Transform (DWT) to segment irrigation cycles. This process is sensitive to precipitation events, and the unpredictable nature of rainfall can affect model performance.

Second, the Kalman filter (KF) observations depend on soil sensor accuracy. Current sensors cannot fully capture spatial heterogeneity, highlighting the need to integrate larger-scale spatial data and additional variables such as evapotranspiration and thermal radiation to improve prediction reliability.

Third, the model primarily uses the irrigation cycle and previous soil moisture values, which is effective for single-step forecasting but insufficient for longer-term multi-step prediction. Capturing more complex dynamics may require extending the set of predictive factors.

Fourth, the nonlinear processes of the irrigation cycle are currently approximated by an averaged autoregressive linear relationship. Future work could explore more complex nonlinear or smoothing functions to further improve model flexibility and predictive performance.

In summary, soil moisture prediction involves multiple interacting factors. Incorporating meteorological variables and crop growth information represents a key direction to overcome current limitations, enhance model robustness, and extend the prediction horizon.

## 4 Conclusions

This study presents a real-time adaptive soil moisture prediction model combining wavelet-based time series segmentation, autoregressive modeling, and Kalman filtering. By decomposing soil moisture data into irrigation-cycle-based subsequences, the approach effectively captures underlying temporal dynamics while mitigating the effects of non-stationarity.

The integration of autoregressive models with a Kalman filtering enables accurate predictions under limited data conditions. A multi-scale sampling strategy further extends the prediction horizon, and the rolling update mechanism allows the model to adapt dynamically to evolving environmental conditions.

Experimental results demonstrate that the proposed model achieves high prediction accuracy for short-term forecasting across multiple soil depths and maintains stable performance under rolling prediction scenarios. Compared with conventional machine learning and deep learning approaches, it provides a favorable balance between accuracy and computational efficiency. Its lightweight design and low data requirements make it particularly suitable for deployment on edge devices in controlled irrigation systems, supporting precision irrigation decision-making.

Despite these advantages, the method has limitations that constrain its applicability. Future work will focus on integrating meteorological variables and crop growth information to enhance generalization and extend the prediction horizon.

## Data Availability

The original contributions presented in the study are included in the article/**Supplementary Material**, further inquiries can be directed to the corresponding author/s.
